# Site fidelity and behavioral plasticity regulate an ungulate’s response to extreme disturbance

**DOI:** 10.1002/ece3.8221

**Published:** 2021-10-28

**Authors:** Samantha E.S. Kreling, Kaitlyn M. Gaynor, Alex McInturff, Kendall L. Calhoun, Justin S. Brashares

**Affiliations:** ^1^ Department of Environmental Science, Policy & Management University of California Berkeley Berkeley California USA; ^2^ School of Environmental and Forest Science University of Washington Seattle Seattle Washington USA; ^3^ National Center for Ecological Analysis and Synthesis University of California Santa Barbara Santa Barbara California USA; ^4^ Bren School of Environmental Science & Management University of California Santa Barbara Santa Barbara California USA

**Keywords:** black‐tailed deer, disturbance, fire ecology, home range, megafire, site fidelity

## Abstract

With rapid global change, the frequency and severity of extreme disturbance events are increasing worldwide. The ability of animal populations to survive these stochastic events depends on how individual animals respond to their altered environments, yet our understanding of the immediate and short‐term behavioral responses of animals to acute disturbances remains poor. We focused on animal behavioral responses to the environmental disturbance created by megafire. Specifically, we explored the effects of the 2018 Mendocino Complex Fire in northern California, USA, on the behavior and body condition of black‐tailed deer (*Odocoileus hemionus columbianus*). We predicted that deer would be displaced by the disturbance or experience high mortality post‐fire if they stayed in the burn area. We used data from GPS collars on 18 individual deer to quantify patterns of home range use, movement, and habitat selection before and after the fire. We assessed changes in body condition using images from a camera trap grid. The fire burned through half of the study area, facilitating a comparison between deer in burned and unburned areas. Despite a dramatic reduction in vegetation in burned areas, deer showed high site fidelity to pre‐fire home ranges, returning within hours of the fire. However, mean home range size doubled after the fire and corresponded to increased daily activity in a severely resource‐depleted environment. Within their home ranges, deer also selected strongly for patches of surviving vegetation and woodland habitat, as these areas provided forage and cover in an otherwise desolate landscape. Deer body condition significantly decreased after the fire, likely as a result of a reduction in forage within their home ranges, but all collared deer survived for the duration of the study. Understanding the ways in which large mammals respond to disturbances such as wildfire is increasingly important as the extent and severity of such events increases across the world. While many animals are adapted to disturbance regimes, species that exhibit high site fidelity or otherwise fixed behavioral strategies may struggle to cope with increased climate instability and associated extreme disturbance events.

## Graphical abstract

Black‐tailed mule deer exhibit high site fidelity, but increase home range size and change habitat selection in response to megafire in northern California.

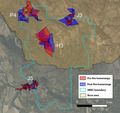

## 1 INTRODUCTION

With rapid global climate change, major disturbance events such as flooding, drought, storms, and wildfires have become more extreme and less predictable (Sergio et al., 2018; Stott, 2016). The ability of wild animals to survive these stochastic events, and to navigate the dramatically altered landscapes that remain, is critical for species persistence. Many contemporary disturbance events do not fall within the range of typical environmental variability experienced over a species’ evolutionary history, and fixed or specialized behavioral strategies may therefore be increasingly maladaptive (; Smith et al., 2021). For example, strong site fidelity may provide fitness benefits in historically stable environments, but trap animals in degraded habitats if major disturbances become common (Abrahms et al., 2017). In contrast, behavioral plasticity may facilitate adaptive responses to novel environmental conditions (van Buskirk, 2012; Xu et al., 2021). An understanding of animal behavior can thus shed light on the mechanisms that might facilitate or impede adaptation to environmental change (Wong & Candolin, 2014).

Nowhere is the challenge of adaptation greater for animals than in the case of modern wildfires. Warming temperatures and changing climate conditions have resulted in wildfires that are historically unprecedented in size and severity (Abatzoglou & Williams, 2016; Abatzoglou et al., 2018; Flannigan et al., 2000; Goss et al., 2020), and the expansion of the wildland–urban interface has increased the frequency of wildfire ignition (Chas‐Amil et al., 2013; Radeloff et al., 2018; Wotton et al., 2003). Though fire plays an integral role in maintaining habitat structure and promoting vegetation growth in many ecosystems, frequent and extreme megafires reduce landscape heterogeneity and biodiversity, with potentially irreversible consequences (Spasojevic et al., 2015). Megafires, generally defined as fires that burn >100,000 acres (405 km^2^; Omi, 2005; Tedim et al., 2018), can move faster and farther and burn hotter and with higher severity than other fires, altering resource distribution, vegetation coverage, landscape morphology, landscape heterogeneity, and soil properties at landscape scales.

While many species have adapted to cope with small, local fires, megafires can create environments with detrimental consequences that may outweigh the benefits that fire typically brings to fire‐adapted landscapes and the animals that live in them (Stephens et al., 2014). Following smaller fires, animals may persist in a burned area and then benefit from the eventual green‐up of new vegetation, or relocate to unburned areas. However, megafires defoliate and burn seed beds in such large areas that individuals may not easily find unburned refugia with sufficient food and shelter resources and vegetation succession may be slowed (Nimmo et al., 2019). In addition, animals with high site fidelity may perceive the risk of leaving their territory or home range to locate unburned patches to be greater than that of remaining in a familiar area with little or no forage (Fagan et al., 2013; Switzer, 1993). While site fidelity can be an adaptive strategy in many instances, this fixed behavioral strategy can also become maladaptive in landscape‐scale disturbances such as megafires where site fidelity may lead to malnourishment and have possible repercussions on survival and fecundity (Abrahms et al., 2017).

Understanding the capacity of ungulate species to adapt to landscapes following megafires is critical to the maintenance of ecosystems and the persistence of local populations of these species. Ungulates play important ecological roles as herbivores and prey (Barbosa et al., 2020), while also influencing functions such as carbon and nutrient cycling, as well as plant regeneration (Forbes et al., 2019). Existing research on the effects of fires on ungulates has primarily focused on the lagged effects of smaller fires after vegetation has regrown (Allred et al., 2011; Rickbeil et al., 2017). Fire can promote growth in vegetation and can cycle nutrients that make forage more nutritious and abundant, leading to “the magnet effect” in which herbivores are attracted to recently burned areas (Allred et al., 2011; Archibald et al., 2005; Cherry et al., 2018). Comparatively, little is known about ungulate behavior during or immediately after a fire event (but see Boyce & Merrill, 1991; Singer et al., 1989), in part due to the difficulty of collecting data during stochastic events.

In 2018, the Mendocino Complex Fire—the third largest fire in recorded California (USA) history—provided a “natural experiment” to study the behavioral responses of ungulates to megafire in a novel before–after control–impact study design. The fire partially burned the University of California's Hopland Research and Extension Center, where we were conducting a study of black‐tailed deer (*Odocoileus hemionus columbianus*) movement and population ecology. As with much of the fauna of the western United States, the fauna of northern California, including black‐tailed deer, evolved in conditions of frequent, small, and cooler fires, but are now experiencing more frequent, larger, and hotter fires (Pausas & Fernández‐Muñoz, 2012; Syphard et al., 2007).

Quantifying the response of black‐tailed deer to megafire is critical for assessing threats to the persistence of this and other ungulates in fireprone ecosystems (Barbosa et al., 2020). Black‐tailed deer exhibit strong site fidelity, which may constrain responses to extreme wildfire at the scale of home range selection (second‐order habitat selection, sensu Johnson, 1980). However, deer have also exhibited fine‐scale behavioral plasticity and flexible habitat use (third‐order habitat selection, sensu Johnson, 1980) in response to many types of large‐scale natural and human disturbances and have thrived in heavily altered environments (e.g., urban settings; Furnas et al., 2020). Deer behavior in the immediate wake of a megafire may therefore provide insights into mechanisms that facilitate animal survival during this period of rapid environmental change and climate volatility (Cherry et al., 2018; Honda et al., 2018; MacDonald‐Beyers & Labisky, 2005).

We explored the following questions: (1) How do deer change their patterns of space use during and immediately after wildfire? (2) How does wildfire influence deer body condition and short‐term survival? To address these questions, we evaluated deer home range size and location, fine‐scale habitat selection, movement trajectories, and body condition before and after the fire, and in burned and unburned areas. We also monitored deer movement and survival during and after the fire event. We expected that deer with home ranges in the burned area would be temporarily displaced by the fire, while those in unburnt patches would remain within their home ranges. We expected deer to select for patches of unburned habitat with greater forage availability, and to avoid burned areas without cover where they would be exposed to greater predation risk immediately post‐fire. We predicted that deer would exhibit more directed movement across burned areas, with greater movement speeds and larger home ranges. We also expected deer with home ranges in the burned area to have declining body condition after the fire compared with deer in the unburned area.

## 2 MATERIALS and METHODS

### 2.1 Study area

**FIGURE 1:**
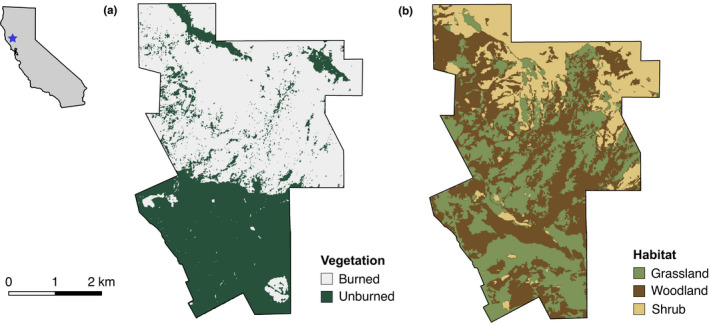
(a) Normalized burn ratio map of burned and unburned vegetation after the Mendocino Complex Fire. (b) Vegetation cover types at the Hopland Research and Extension Center. Inset shows location of study area in California

We conducted our fieldwork at the University of California Hopland Research and Extension Center (HREC), located in southern Mendocino County (39°00′N, 123°04′W; Figure 1). The 21.4‐km^2^ study area is situated at the wildland–urban interface, a key zone of interest for fire science, and is bordered to the south by development (a town and major highway) and to the north by protected wildlands. The study area is comprised of a heterogeneous mixture of habitat types, including chaparral/shrubland, oak woodland, and grassland, and deer in the study area access resources in all of these habitats. The region has a Mediterranean climate, with mild seasons and winter rains. Topography of the study area is characterized by rugged inclines and several ravines through which water drains in the wet season. The property has a number of agricultural pastures with low fences that deer can easily cross, and the deer population is free‐ranging.

In 2018, California had its worst fire season in recorded history at the time of the study (surpassed in 2020), in terms of area burned, with 8527 fires burning nearly 7700 km^2^ (NIFC, 2018). On July 27, 2018, the Mendocino Complex Fire broke out north of the study area (Costafreda‐Aumedes et al., 2018). Between the date of ignition and September 18, 2018, the fire burned a total of 1858 km^2^, becoming the second largest fire in California history. On July 27, the Mendocino Complex Fire entered HREC, burning the study area until July 28. Up to eight weeks after the initial event, trees continued to smolder, and small fires emerged. The fire burned roughly 65% of HREC (13.8 km^2^), with burns concentrated in the northern half of the study area across a range of different habitat types including oak (*Quercus* spp.) woodlands, madrone (*Arbutus menziesii*) forests, manzanita (*Arctostaphylos* spp.) shrubland, and grasslands (Figure 1). We treated the Mendocino Complex Fire as a natural experiment, and our study design was therefore constrained by the data collection methods already in place at the time of the fire.

### 2.2 Monitoring deer movement

To monitor movement of black‐tailed deer in the study area, we deployed GPS collars on 18 adult deer between July 2–19, 2018, including 16 female deer and 2 male deer (Table S1). Deer were captured using Clover traps and were manually restrained without the use of chemical immobilizers. Of the 18 collared deer, 13 had home ranges within the burn perimeter, facilitating the comparison of deer movement in burned and unburned areas.

We used Vectronic VERTEX Plus collars and Lotek Iridium Track M collars for female deer, and ATS Iridium Lite G2110L expandable collars for male deer. Vectronic collars recorded GPS locations every hour, and ATS collars, every two hours. We chose to use a longer fix rate for the ATS collars on males to maximize collar life span, given the difficulty of capturing male deer (they less readily enter Clover traps, which accounts for the smaller sample size of males in our study). We remotely monitored deer for multiple days after capture to ensure there were no lasting negative effects from handling without interrupting deer behavior.

To compare deer movement before and after the fire, we subset our data such that there was the same number of pre‐ and post‐fire GPS data points for each individual in the study. This resulted in pre‐ and post‐fire periods ranging from 15 to 25 days, depending on how many days before the fire a given individual had been collared. For all analyses of GPS collar data, we removed the first 24 h of post‐capture data to ensure paths were representative of typical behavior. We believe that this cutoff is reasonable, as visual inspection revealed that deer resumed normal activity within hours of release. We also removed three erroneous GPS locations from the dataset, given that they were far from the study area with no nearby consecutive points within 2 km.

### 2.3 Home ranges and displacement

We used the local convex hull method (LoCoH) to determine home range size (Getz et al., 2007). We calculated 95% isopleths for each individual for the pre‐ and post‐fire periods using the T‐LoCoH and adehabitatHR packages in R (Calenge, 2006; Lyons, 2018; Lyons & Getz, 2018). We used a *k*‐nearest neighbors approach with *k* = 15 neighbors, which we determined to be an acceptable *k* value for all individuals based on isopleth area curves and isopleth area–edge ratio plots (Dougherty et al., 2018). When calculating isopleth area, we did not consider a temporal effect (*s* = 0). We used paired Welch's unequal variance t tests to compare deer home range size before and after the fire for female deer only, given that male deer had significantly larger home ranges than female deer, and a low sample size prevented an independent analysis of males (we instead report summary metrics for the male deer).

To determine the displacement distance of deer as a result of the fire, we identified the point during the fire and the 3‐day post‐fire period that was farthest from the pre‐fire LoCoH home range centroid and calculated the Euclidean distance between points. We calculated the distance between the centroids of pre‐ and post‐fire isopleths for each deer to examine whether, and how far, deer shifted their home ranges after the fire. We calculated displacement for all 18 deer collared pre‐fire, including those with home ranges inside and outside of the fire perimeter.

### 2.4 Movement metrics

We calculated pre‐fire and post‐fire movement metrics for each individual deer, including average step length, mean turn angle correlation (TAC), mean time to return (hours an animal spends before returning to a given radius), and mean residence time (number of hours spent inside a given radius), using the *amt* package in R (Signer, 2018). The radius was set equal to mean step length, following Abrahms et al. (2017). To understand whether deer movement became more directed after fire, we calculated the straightness index, a measure of path tortuosity that ranges from 0 to 1, where 1 represents perfect linearity between distance and trajectory length (Benhamou, 2004). After initial exploration suggested differences in TAC, mean time to return, and mean residence time between males and females, we excluded males from the analysis (a low sample size prevented an independent analysis of male deer, and the fix rate of the collars was different for males and females).

### 2.5 Resource selection functions

We used resource selection functions (RSFs) to examine patterns of deer habitat selection before and after the fire, for the deer with home ranges in the burn perimeter (*n *= 13). We generated 4 random points for each GPS location for each deer within the 95% minimum convex polygon corresponding to the combination of their pre‐ and post‐fire home ranges. We used the *lme4* package in R to run logistic regressions (GLMMs; Bates et al., 2015). Given small sample sizes of male deer, we combined male and female deer in RSF models, and explored the effect of sex as a fixed effect in model selection. We modeled pre‐ and post‐fire time periods separately.

We used a hypothesis‐driven approach to select covariates that we believed to influence deer movement, based on our understanding of the study system and on previous studies of black‐tailed deer in the region (Bose et al., 2018; Table S2). The covariates we considered in the RSFs were sex, vegetation type, elevation, slope, aspect (northness and eastness), ruggedness, distance to streambed, and surviving vegetation (post‐fire model only). We confirmed that variance inflation factor (VIF) <3 for all covariates, a common cutoff for multicollinearity (O’Brien, 2007). All covariates were standardized prior to running the model. We then used an information‐theoretic approach to model selection, using a backward stepwise approach from the full model and selecting the best model based on AIC (Burnham & Anderson, 2002).

To create the vegetation‐type layer, we hand‐digitized vegetation classes from high‐resolution (<1 m) National Agriculture Imagery Program aerial imagery (2014–2015) to create a vegetation classification layer of the study area. In 2015, we ground‐truthed the vegetation classification for the entire HREC study area, visiting 50 random points and validating their digital classification (accuracy was 98%). For our analyses, we simplified land cover classes into three categories: shrubland (chaparral), woodland, and grassland.

We obtained elevation and slope data from the ASTER Global Digital Elevation Model (NASA & METI, 2011). We derived aspect from these DEM data and calculated northness (cosine of the aspect layer) and eastness (sine of the aspect layer). We also calculated ruggedness, which considers variability in both slope and aspect within a neighborhood of 2500 m^2^ using the Vector Ruggedness Measure tool for ArcGIS, which was adapted from Hobson (1972). We created a raster layer of distance from streambeds (seasonal streams, which were mostly dry during the study period) on the study site. We obtained stream vector data from the National Hydrography Dataset and calculated the distance from any given cell in the raster to the nearest stream. Finally, we created a layer of post‐fire surviving vegetation using the near‐infrared and shortwave infrared bands from 3‐m resolution satellite imagery acquired on August 8, 2018, five days post‐fire by calculating the normalized burn ratio (NBR; Imagery courtesy of Planet Labs, Inc.). Positive NBR values were classified as vegetated (value 1), and negative NBR values were classified as burnt (value 0; Escuin et al., 2007).

To assess the predictive ability of the models, we validated the top models using area‐adjusted cross‐validation, following Boyce et al. (2002). We ran 1300 bootstrapped iterations with replacement in which we randomly subset the data, training the model on 80% of the data and withholding 20% for testing. We ran 100 iterations for each of the 13 deer, separating the deer for the area‐adjusted cross‐validation given differences in available habitat in each deer's home range. For each iteration, we divided the study area (the given deer's MCP home range) into 10 bins based on deciles of predicted risk for the test data and calculated the Spearman rank coefficient between bin rank and the mean area‐adjusted frequency of deer locations from the test data, for all iterations across deer combined.

### 2.6 Assessing deer body condition

We used images from camera traps to assess the effects of wildfire on deer body condition. Beginning in 2016, we deployed a grid of 36 motion‐activated Reconyx Hyperfire PC900 and HC600 infrared cameras (Figure S1). We placed each camera trap at the centroid of a hexagonal grid cell, spaced 750 m apart from cameras in the six neighboring grid cells (the area of each grid cell was 0.37 km^2^). To facilitate comparison across camera sites, we placed cameras at the most suitable location within 50 m of the predetermined grid cell center to maximize detection probability, by facing game trails, for example. Cameras were unbaited and mounted 1 meter high in steel cases on trees, or on steel posts when there were no trees nearby.

Of the 36 cameras, 25 cameras were in burned areas within the Mendocino Complex Fire perimeter, and 11 cameras were in unburned areas (Table S3). Memory cards in six cameras in the burn area were not salvageable due to fire damage and excluded from the analysis (*n *= 6). We additionally excluded cameras that were operational for <40 days during either the pre‐ or post‐fire time period. Five of the cameras were non‐functioning after the fire, but we recovered data from the memory cards and replaced the cameras between August 1 and August 8 with Bushnell Trophy Cams. An additional 3 cameras inside the fire perimeter did not capture any deer photographs suitable for estimating body condition index (BCI) from. One camera outside of the burned area was also excluded due to vegetation blocking the camera pre‐fire. Otherwise, all cameras in the burned and unburned areas were operating continuously before, during, and after the fire. This resulted in a total of 10 cameras outside of the burn area and 15 cameras inside the burn area.

Following Smiley (2017), we categorized records of adult male and female deer from each camera into a BCI. BCI values range from 0 to 5 based on the visibility of five bone regions (scapula, spinal ridge, ribs, tuber ischium, and tuber ilium) and are correlated with subcutaneous fat storage (see full details in Smiley, 2017). Each photograph was reviewed by one person. We defined independent camera records as those that occurred at least 15 min after the previous record. We removed photographs from analysis if more than 60% of the deer's body was not visible due to lighting, picture quality, or deer position. We were unable to identify individual deer at the camera traps, but we know that the study area hosts a high density of deer. We designed the camera grid such that each grid cell was larger than an individual deer's home range, and we believe that the camera traps were set far enough apart to limit amount of resampling the same individuals based on home range size (typical home range size: 0.1–0.3 km^2^; camera grid cell: 0.37 km^2^).

We then compared body conditions of deer pre‐ and post‐fire, inside and outside (control group) of the burn perimeter. We defined the 60‐day pre‐fire period as June 1–July 27, 2018, and the 60‐day post‐fire period as July 28–September 30, 2018 (120 total days). We chose these time periods to be long enough to capture a representative sample of animal activity, but not so long that seasonal influences could greatly affect our results. We then used linear regression models to evaluate BCI as a function of whether the observation was inside or outside of the burn perimeter, time period (before or after fire), days since fire (set to 0 for all pre‐fire observations), and interaction terms (between burn and time period, and between burn and days since fire). We did not include time period and days since fire in the same models due to collinearity. We included camera location as a random effect to account for the possibility of resampling individuals. We compared models using Akaike's information criterion (AIC) and evaluated model fit using the *MuMIn* package in R to calculate conditional pseudo‐*r*‐square (Barton, 2018). To evaluate potential spatial autocorrelation in BCI across camera sites, we calculated Moran's I for mean BCI for male and female deer across cameras in the pre‐ and post‐fire periods, using the *ape* package in R (Paradis & Schliep, 2018).

## 3 RESULTS

### 3.1 Home ranges and displacement

**FIGURE 2:**
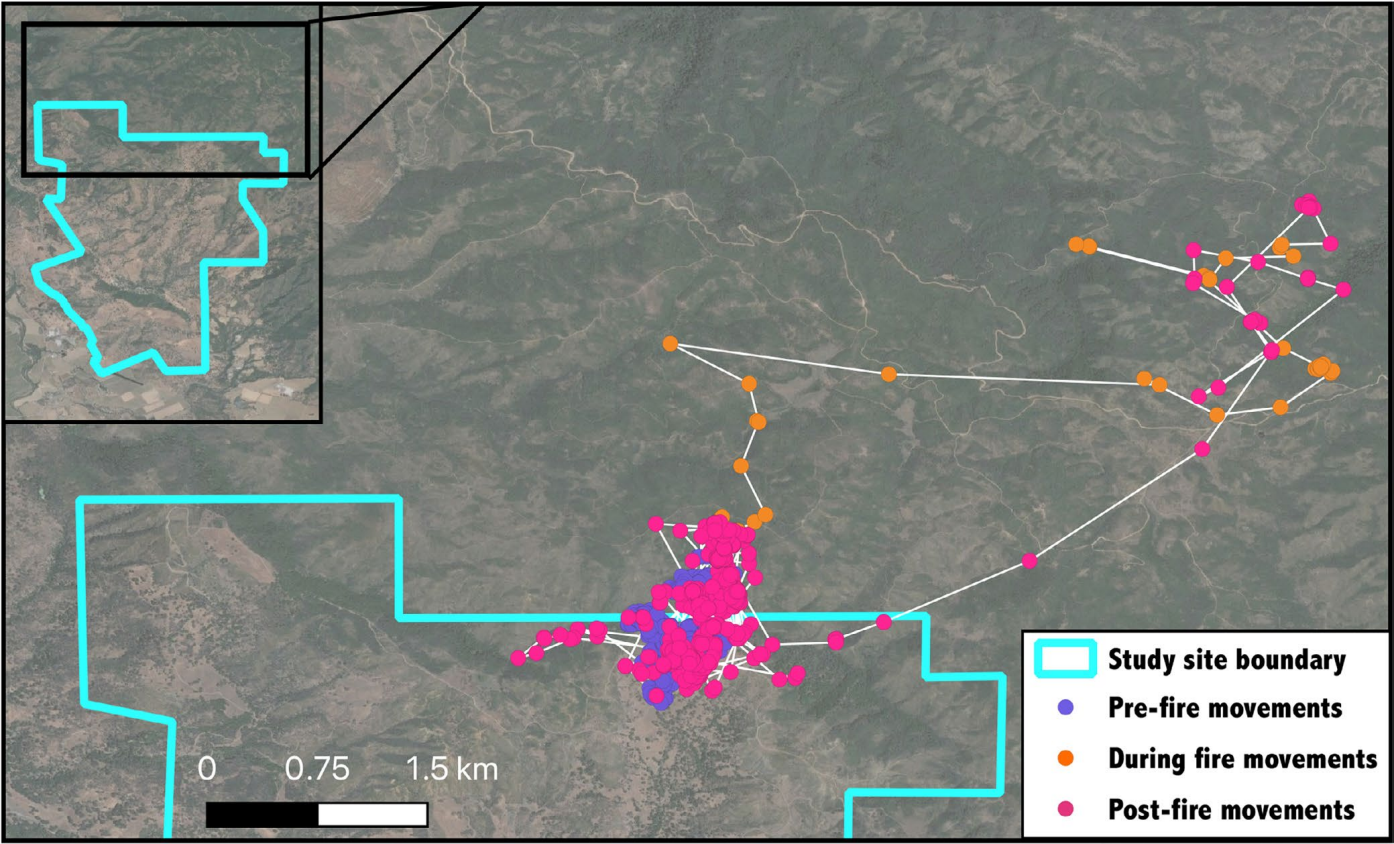
Example of deer displacement during the Mendocino Complex Fire. Female deer J3 left her pre‐fire home range at the northern border of the Hopland Research and Extension Center study area (purple points; July 4–July 26, 2018), traveling over 4 km in front of the flames (orange points; July 27–August 3, 2018), then promptly returned to the same home range after the fire (pink points; August 4–August 21, 2018). Points correspond to hourly GPS fixes

The Mendocino Complex Fire displaced deer with home ranges within the fire perimeter (*n* = 13) an average of 1.17 km (standard deviation = 1.11 km, range: 0.042–4.4 km) from their home range centroid points (Figure 2). In comparison, deer with home ranges outside of the burn perimeter (*n* = 5) had a significantly lower mean displacement of 0.25 km (standard deviation = 0.11 km, range: 0.13–0.43 km) during the fire event (*t* = 2.97, *p* = .01, *df* = 12.64).

**FIGURE 3:**
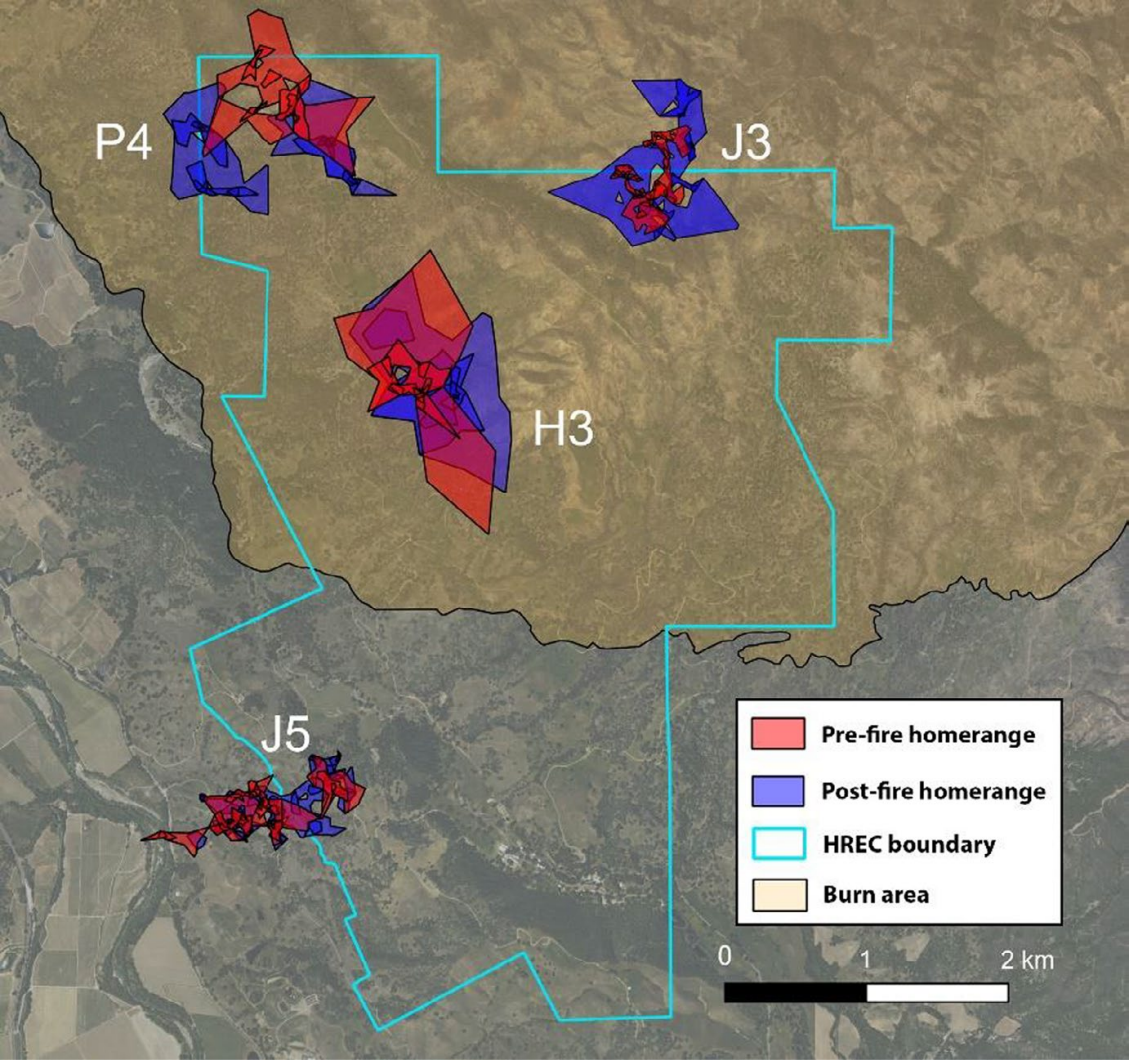
Home ranges of four collared deer (males H3 and P4, and females J3 and J5) in the Hopland Research and Extension Center study area in Hopland, California. The map depicts LoCoH home ranges for each deer before the Mendocino Complex Fire (from early‐mid July capture date through July 27, 2018) and immediately after the fire (for the same number of days as the pre‐fire period, for each deer). To facilitate visualization, we selected four of the 18 collared deer to exemplify home ranges in non‐overlapping regions of the study area, with J5 as a comparison for deer outside of the fire perimeter

Following the fire, the home range size of female deer in the burned area (*n* = 11) increased by an average of 41%, with a pre‐fire mean home range size of 0.17 km^2^ (SD ± 0.05) compared with 0.24 km^2^ post‐fire (SD ± 0.11; *t* = −1.97, *df* = 10, *p* = .08). In comparison, home range size did not change meaningfully for nearby female deer outside of the burn perimeter (*n* = 5; pre‐fire home range size mean = 0.14 km^2^, SD ± 0.09; post‐fire mean = 0.20, SD ± 0.14; *t* = −1.43, *df* = 4, *p* = .25). Mean home range size for the two collared male deer in the burn area more than doubled from 0.38 to 0.78 km^2^ (an increase of 0.32 km^2^ for P4 and 0.44 km^2^ for H3; illustrated in Figure 3), although the small sample size precluded tests of statistical significance.

While home range size increased after the fire, the location of the home ranges of individual female deer did not change significantly when compared to female deer outside of the burn perimeter (*t* = 0.40, *p* = .70, *df* = 5.49). On average, the centroid of female deer home ranges (*n* = 11) within the fire shifted by 140.32 m (SD = 67.97 m), and the two male deer home ranges shifted by 30.13 and 36.10 m. Female deer outside of the burn perimeter (*n* = 5) shifted their home ranges on average by 119.22 m (SD = 108.37).

### 3.2 Movement metrics

Female deer in the burned area traveled a significantly greater distance per day after the fire, approximately 1.5 times the daily distance traveled before the fire (Table 1). Path straightness also increased after the fire, but not significantly (Table 1). Additionally, mean residence time significantly increased. There was no significant difference in pre‐ and post‐fire mean turn angle correlation or mean time to return (Table 1). No *t* tests showed any significant change in movement metrics for female deer outside of the burn perimeter (Table S4).

**TABLE 1:** Comparison between pre‐ and post‐fire home range and movement measures for female black‐tailed deer with home ranges in the burned area of the Hopland Research and Extension Center (*n* = 11)

Movement metric	Pre‐fire (mean ± SD)	Post‐fire (mean ± SD)	*df*	*t*	*p*‐Value
*Home range size (km^2^)*	*0.17 ± 0.05*	*0.24 ± 0.11*	*10*	*−1.97*	.*08*
**Daily movement distance (km)**	**1.47 ± 0.26**	**2.15 ± 0.37**	**10**	**−5.68**	**<.001**
*Straightness index*	*0.011 ± 0.01*	*0.021 ± 0.02*	*10*	*−2.06*	.*07*
Turn angle correlation	0.515 ± 0.02	0.5202 ± 0.03	10	−0.55	.60
**Mean residence time (min)**	**10.0 ± 4.2**	**16.8 ± 10.6**	**10**	**−2.51**	.**03**
*Mean time to return (min)*	*64.1 ± 16.1*	*55.5 ± 12.7*	*10*	*2.02*	.*07*

Note

Bold values represent differences at the significance level α = .05, and italicized values represent differences at the significance level α = .10.

### 3.3 Resource selection functions

For both the pre‐fire and post‐fire periods, the best RSF model for deer with home ranges in the burned area was the full model, with all covariates: vegetation type, elevation, slope, aspect, ruggedness, and distance to streambed, and surviving vegetation (post‐fire model only; Table S5). Deer selected for similar topographic features before and after the fire, including higher elevation, more rugged areas, flatter slopes, and east‐ and north‐facing slopes (Table 2). Deer also selected for areas farther from streambeds before and after the fire.

**TABLE 2:** Beta‐coefficients and standard errors for all coefficients in the top Resource Selection Function models for deer before and after the Mendocino Complex Fire at the Hopland Research and Extension Center

Variable	Pre‐fire			Post‐fire		
	Beta‐coefficient	Standard error	*p*‐Value	Beta‐coefficient	Standard error	*p*‐Value
Intercept	−1.524	0.122	<.001	−0.816	0.573	.015
Shrubland vegetation type	0.028	0.036	.424	−0.901	0.042	<.001
Grassland vegetation type	−0.175	0.046	.001	−0.884	0.055	<.001
Elevation	0.238	0.041	<.001	0.267	0.042	<.001
Slope	−0.148	0.019	<.001	−0.064	0.021	.002
Aspect (East)	0.050	0.018	.005	0.134	0.020	<.001
Aspect (North)	0.132	0.017	<.001	0.114	0.019	<.001
Streambed distance	0.080	0.022	<.001	0.082	0.022	.002
Ruggedness	0.063	0.027	.018	0.320	0.030	<.001
Surviving vegetation	NA	NA	NA	0.739	0.036	<.001

Note

Vegetation type was a factor, and woodland was the reference level. Surviving vegetation was also a binary factor, and “no surviving vegetation” was the reference level. All models included deer ID as a random effect.

Before the fire, deer exhibited weak vegetation‐type preferences. They selected for woodland, exhibited no selection for chaparral, and avoided grassland (Table 2). After the fire, in contrast, deer preferred woodland and avoided grassland and shrubland. Vegetation‐type preferences were much stronger after the fire than before the fire. Furthermore, after the fire, deer selected strongly for unburned areas with surviving vegetation (for deer with home ranges in the fire perimeter, a total of 29% of the area within deer post‐fire home ranges were unburned; Table 2).

Our model validation suggests that the pre‐fire and post‐fire models were strongly predictive of deer landscape use (pre‐fire, all deer combined: *r_s_* = .98, *p* < .0001; post‐fire, all deer combined: *r_s_* = .99, *p* < .0001).

### 3.4 Assessing deer body condition

Before the fire, most deer had a body condition index (BCI) between 2 and 3, where scapula and ribs were visible to somewhat visible, but spinal ridge was not. However, after the fire and predominantly within the burned area, some deer were sighted with a BCI of 0, wherein all 5 body markers used to determine BCI were clearly visible, implying a very low level of body fat.

Female deer BCI declined in burned areas after the fire, while male deer BCI did not, as revealed by the top models (lowest AIC). The best model for female deer BCI was the full model, including the interaction between burn (inside vs. outside) and time period (conditional pseudo‐*r* ^2^ = .24; Tables S6, S7). Within the burn area, female deer BCI was lower after the fire (*n* = 273 camera trap detections, mean = 2.53 ± SD 0.98) than before the fire (*n* = 325 camera trap detections, mean = 3.10 ± SD 0.82). In comparison, there was no difference in pre‐ and post‐fire BCI for the female deer outside of the fire perimeter (*n* = 370 and 199 camera trap detections, respectively). Male deer BCI (*n* = 172 pre‐fire inside, 45 post‐fire inside, 130 pre‐fire outside, and 39 post‐fire outside camera trap detections) did not differ between burned and unburned areas, nor in the pre‐ and post‐fire periods (null model was top model, conditional *R* ^2^ = .24; Table S6). There was no evidence that mean BCI values were spatially correlated (female pre‐fire: Moran's *I* = <0.001, *p* = .21; female post‐fire: Moran's *I* = −0.04, *p* = .93; male post‐fire: Moran's *I* = −0.10, *p* = .55), with the exception of male deer pre‐fire BCI, which showed some evidence of spatial autocorrelation (Moran's *I* = 0.04, *p* = .04).

## 4 DISCUSSION

The immediate responses of individual animals to extreme disturbances can have important consequences for the recovery and persistence of populations and communities. A megafire in northern California, USA, in 2018 provided a unique natural experiment that shed light on the role of animal behavior in mediating responses to disturbance. Strong site fidelity constrained responses of individual animals at the scale of the home range, and poorer body condition was associated with female deer within the burn areas. However, behavioral plasticity with regard to movement and habitat use within home ranges facilitated animal survival in the wake of extreme disturbance.

During the Mendocino Complex Fire, black‐tailed deer at the Hopland Research and Extension Center fled the megafire and survived, but quickly returned to their original home ranges. Our fine‐scale examination of deer movement patterns, resource selection, and space use revealed that deer adjusted their behavior to adapt to a burned and depleted environment. Despite a decline in female body condition after the fire, all individuals in our study survive the five‐month study period after the fire, during a time of the year when resources are most limited. Thus, adaptive capacity granted by behavioral plasticity provided an important buffer for coping with shifting environmental conditions (Gross et al., 2010; Hammond et al., 2018).

Deer exhibited strong site fidelity to their small home ranges, which constrained their spatial response to the fire. All deer quickly returned to and remained in their pre‐fire home ranges despite dramatic landscape changes and reduced forage, even while there was high‐quality, unburned forage on average 1.6 km away. Our findings contrast predictions that ungulates and other large mammals in California shrubland systems flee to areas outside of the burn perimeter and remain there until the habitat is suitable for recolonization (van Mantgem et al., 2015). Although high site fidelity can have potential benefits of reducing competition and predation risk given site familiarity (Forrester et al., 2015), high site fidelity may become maladaptive as climate change increases the severity and frequency of extreme events, compromising survival (Abrahms et al., 2018). The declining body condition of female deer in the burned areas as compared to those in the unburned areas attests to the cost of living within the burn scar. Other studies have linked environmental disturbance to reduced fecundity and offspring health (McHuron et al., 2018; Sapolsky et al., 2000), and it is possible that deer in our study experienced fitness consequences despite surviving the disturbance.

While high site fidelity may constrain the ability of an individual animal to relocate following disturbance events, deer adjusted their behavior in an effort to meet foraging demands despite resource scarcity. Deer doubled their home range size and increased daily movement, likely in response to reduced resource availability in their home ranges. Female deer also increased their mean residence time after the fire, suggesting that they engaged in less exploratory behavior, and spent more time in the fewer remaining areas of shelter and forage. There was weak evidence to suggest that deer movements were straighter and more directed after the fire (Table 1), possibly because deer exhibited directed movement between these remaining vegetation patches. Additionally, removal of vegetation likely facilitated more direct movement through the landscape and may have decreased the risk of predation from mountain lions (ambush predators that rely on cover to hunt), emboldening deer (Hopcraft et al., 2005; Jaffe & Isbell, 2009).

In addition to increasing space use and changing movement patterns, deer changed their patterns of habitat selection within their home ranges in response to the altered landscape. Before the fire, deer exhibited weak habitat preferences, while after the fire, they strongly selected for woodland habitat, avoiding the shrubby areas that had largely burned. These wooded areas provided shelter from the elements and cover from non‐ambush predators, while the chaparral shrub burned at high severity, leaving little cover (Wilkin et al., 2017). Before and after the fire, deer selected for areas farther from the dry streambeds, possibly because the drainages throughout the study area facilitate predator movement and may therefore be avoided by deer.

Irrespective of habitat type, the RSF analysis revealed that deer also strongly selected for the small islands of surviving vegetation post‐fire within the study area, likely relying on these patches of vegetation for forage and shelter. Our findings suggest that unburned patches not only serve as important post‐fire refuges for animals with smaller home ranges, such as rodents (Pereoglou et al., 2011) and birds (Lindenmayer et al., 2008), but may also facilitate a landscape supplementation strategy for species with larger home ranges that move between these unburned patches (Nimmo et al., 2019). Threats to species imposed by widespread homogenization of landscapes from megafire are already being witnessed in other parts of the United States, Australia, and the Amazon (Brando et al., 2020; Pickrell & Pennisi, 2020). Generalist species and other larger‐bodied animals, similar to black‐tailed deer, may be less impacted by isolated megafire events, but repeated events that lead to permanent habitat conversion may exhaust the adaptive capacity of these species. Maintaining habitat heterogeneity and access to preferred vegetation patches may therefore be an important conservation consideration for deer and other species impacted by recent fires. Proper fire management before megafires occur, and recovery programs that reseed lost plant species post‐fire are key strategies currently being used across the world to help maintain landscape heterogeneity after these extreme events occur (Wintel et al., 2020).

Although we recognize the limitations of inference based on this one‐time natural experiment, our study contributes to a growing body of literature on the role of behavior in mediating animal responses to disturbance (Sih et al., 2011). As the frequency, size, and severity of wildfires and other disturbances become greater and more widespread as a result of global climate change, behavioral plasticity may become the deciding factor between survival and extinction of populations (Muñoz et al., 2015). Animals may survive by changing their movement, diet, and intraspecific interactions, or shifting life events such as migration or reproduction (Cohen et al., 2018; Grazer & Martin, 2012; Wong & Candolin, 2014). Meanwhile, fixed behavioral strategies such as high site fidelity may become maladaptive amidst increasing disturbance (Abrahms et al., 2018; Muñoz et al., 2015). While small sample sizes impeded our ability to explore individual variation or tease apart sex differences in this study, behavioral syndromes may shape inter‐individual variation in responses to disturbance, and disturbance may thus drive selection for greater plasticity (Sih et al., 2004). Future research should consider the trade‐offs between behavioral plasticity and fixed behavioral strategies such as site fidelity in the wake of large disturbance events and in the face of climate change. By studying these responses, we may also gain insight on interventions, such as food or cover augmentation, that will be critical to fostering resistance and recovery of affected animals.

## 5 CONCLUSION

The Mendocino Complex Fire was the second largest fire in California history yet elicited surprisingly few behavioral responses from deer, which remained in severely burned home ranges. High site fidelity is likely an underreported risk factor in the face of extreme events and variability under climate change. The behavioral adjustments we did observe, however, may have facilitated the survival of deer following this extreme environmental disturbance. While we have shown how behavioral plasticity allows deer to alter their foraging patterns to adapt to a changed landscape, additional research in response to other types of disturbance events is important to further understand how animals will cope with ongoing global change.

## CONFLICT OF INTEREST

No authors have any conflicts of interest to report.

## AUTHOR CONTRIBUTION

**Samantha E.S. Kreling:** Conceptualization (equal); Data curation (equal); Formal analysis (equal); Methodology (equal); Writing‐original draft (equal); Writing‐review & editing (equal). **Kaitlyn M. Gaynor:** Conceptualization (equal); Data curation (equal); Formal analysis (equal); Methodology (equal); Validation (lead); Writing‐original draft (equal); Writing‐review & editing (equal). **Alex McInturff:** Conceptualization (equal); Methodology (equal); Writing‐original draft (equal); Writing‐review & editing (equal). **Kendall L. Calhoun:** Conceptualization (equal); Writing‐original draft (equal); Writing‐review & editing (equal). **Justin S. Brashares:** Conceptualization (equal); Methodology (equal); Supervision (equal); Writing‐original draft (equal); Writing‐review & editing (equal).

### OPEN RESEARCH BADGES

This article has earned an Open Data Badge for making publicly available the digitally‐shareable data necessary to reproduce the reported results. The data is available at https://doi.org/10.5061/dryad.x0k6djhjg.

## Supporting information

Supplementary Material

## Data Availability

Data used in this study can be found on Dryad (https://doi.org/10.5061/dryad.x0k6djhjg ).
