# Supplementary material for: Site fidelity and behavioral plasticity regulate an ungulate’s response to extreme disturbance
Source: Ecol Evol. 2021 Oct 28;11(22):15683–94. doi: 10.1002/ece3.8221 (PMC8601917; doi:10.1002/ece3.8221)
Supplement: Supplementary file 1 — Supplementary Material [file ECE3-11-15683-s001.docx]

**SUPPLEMENTARY MATERIALS**

**Supplementary Table 1:** Information on GPS-collared deer at the Hopland Research and Extension Center.

| **Deer ID** | **Inside Burn Perimeter (Y/N)** | **Sex**  **(M/F)** | **Date Collared** | **Length of Pre-Fire and Post-Fire Periods** | **Number of GPS Locations per Period** |
| --- | --- | --- | --- | --- | --- |
| A4 | Y | F | 7/19/2017 | 27 | 648 |
| A5b | N | F | 7/3/2018 | 24 | 576 |
| D3b | Y | F | 7/2/2018 | 25 | 600 |
| F2 | Y | F | 7/14/2018 | 13 | 312 |
| H2 | Y | F | 7/2/2018 | 25 | 600 |
| H3 | Y | M | 7/10/2018 | 17 | 204 |
| H4 | N | F | 7/12/2018 | 15 | 360 |
| H5 | N | F | 7/12/2018 | 15 | 360 |
| I5 | Y | F | 7/3/2018 | 24 | 576 |
| J1 | Y | F | 7/3/2018 | 24 | 576 |
| J2 | Y | F | 7/3/2018 | 24 | 576 |
| J3 | Y | F | 7/3/2018 | 24 | 576* |
| J5 | N | F | 7/3/2018 | 24 | 576 |
| K1 | Y | F | 7/5/2018 | 22 | 528 |
| K2 | Y | F | 7/5/2018 | 22 | 528 |
| K4 | N | F | 7/19/2018 | 8 | 192 |
| P4 | Y | M | 7/2/2018 | 25 | 300 |
| Q5 | Y | F | 7/2/2018 | 25 | 600 |

** We removed the first 54 post-fire points for J3, as they did not characterize typical home range behaviour (this deer was displaced from its home range for an extended period of time after the fire). All of the other deer returned to their home ranges immediately after the fire (or never left their home ranges).*

**Supplementary Table 2:** Hypothesized relationships between environmental covariates and deer habitat selection, before and after the Mendocino Complex Fire at the Hopland Research and Extension Center.

| **Covariate** | **Hypothesis** |
| --- | --- |
| Aspect | We expected deer to select for northern and western-facing slopes as refugia from the summer heat, and greater availability of nutritional forb-like vegetation (both pre- and post-fire) |
| Distance to stream bed | We expected deer to select for locations away from stream beds due to higher predation risk, given that most streambeds were dry during the study period (both pre- and post-fire) |
| Elevation | We expected deer to select for areas of higher elevation, given findings of previous studies of black-tailed deer (Bose et al. 2018; (both pre- and post-fire) |
| Ruggedness | We expected deer to select for less rugged terrain, given that rugged terrain is associated with higher risk of predation and movement requires greater energy expenditure (both pre- and post-fire) |
| Slope | We expected deer to select for areas with lower slope as these areas are less energetically costly and represent different predation risk costs. |
| Surviving vegetation | We expected deer to select for areas of surviving vegetation (post-fire only), which provide forage and shelter |
| Vegetation type | Given that deer are habitat generalists, we expected deer to select for all vegetation types equally (pre-fire). After the fire, we expected to see greater selection for woodland than grassland or shrubland, given that they provided the only shelter after the grass and shrubs burned |

**Supplementary Table 3:** Information on camera trap operation and associated deer observations for Body Condition Index analysis, before and after the Mendocino Complex Fire at the Hopland Research and Extension Center.

| **Camera** | **Inside Burn Perimeter** | **Destroyed in Fire** | **Length of pre-fire period (days)** | **Length of post-fire period (days)** | **Included in BCI** | **Number of Doe observations pre-fire** | **Number of Doe observations post-fire** | **Number of Buck observations pre-fire** | **Number of Buck observations post-fire** | **Notes on reasons for removal** |
| --- | --- | --- | --- | --- | --- | --- | --- | --- | --- | --- |
| A03 | Y | Y | NA | NA | N | NA | NA | NA | NA |  |
| A13 | N | N | 60 | 60 | Y | 2 | 1 | 1 | 0 |  |
| B02 | Y | N | 52 | 54 | Y | 16 | 25 | 70 | 3 |  |
| B04 | Y | N | NA | NA | N | 0 | 0 | 0 | 0 | Did not have any deer observations that were suitable for BCI analysis |
| B06 | Y | N | NA | NA | N | 0 | 0 | 0 | 0 | Did not have any deer observations that were suitable for BCI analysis |
| B08 | Y | N | NA | NA | N | 0 | 0 | 0 | 0 | Did not have any deer observations that were suitable for BCI analysis |
| B10 | Y | N | 60 | 60 | Y | 3 | 0 | 3 | 0 |  |
| B12 | N | N | 60 | 60 | Y | 0 | 12 | 1 | 3 |  |
| B14 | N | N | 60 | 60 | Y | 313 | 86 | 117 | 31 |  |
| C03 | Y | N | 60 | 60 | Y | 25 | 29 | 2 | 3 |  |
| C05 | Y | Y | NA | NA | N | NA | NA | NA | NA |  |
| C07 | Y | Y | NA | NA | N | NA | NA | NA | NA |  |
| C09 | Y | N | 60 | 60 | Y | 22 | 8 | 0 | 2 |  |
| C11 | Y | Y | NA | NA | N | NA | NA | NA | NA |  |
| C15 | N | N | 60 | 60 | Y | 2 | 5 | 1 | 2 |  |
| C17 | N | N | 60 | 60 | Y | 3 | 9 | 0 | 1 |  |
| D04 | Y | N | 60 | 60 | Y | 55 | 2 | 6 | 0 |  |
| D06 | Y | N | 60 | 56 | Y | 11 | 4 | 3 | 0 |  |
| D08 | Y | N | 60 | 60 | Y | 12 | 4 | 28 | 4 |  |
| D10 | Y | Y | NA | NA | N | NA | NA | NA | NA |  |
| D12 | N | N | 60 | 60 | Y | 14 | 21 | 0 | 0 |  |
| D14 | N | N | 60 | 60 | Y | 19 | 29 | 0 | 0 |  |
| D16 | N | N | 60 | 60 | Y | 4 | 17 | 3 | 0 |  |
| E05 | Y | N | 60 | 60 | Y | 71 | 80 | 18 | 9 |  |
| E07 | Y | N | 60 | 60 | Y | 9 | 3 | 0 | 1 |  |
| E09 | Y | N | 60 | 60 | Y | 24 | 33 | 0 | 0 |  |
| E11 | Y | N | 60 | 60 | Y | 5 | 4 | 7 | 3 |  |
| E13 | N | N | 60 | 60 | Y | 7 | 12 | 5 | 2 |  |
| E15 | N | N | 60 | 60 | Y | 6 | 7 | 2 | 0 |  |
| E17 | N | N | NA | NA | N | NA | NA | NA | NA | Grass blocking camera pre-fire |
| F04 | Y | Y | NA | NA | N | NA | NA | NA | NA |  |
| F06 | Y | N | 60 | 51 | Y | 6 | 2 | 0 | 0 |  |
| F08 | Y | N | 60 | 60 | Y | 27 | 43 | 1 | 1 |  |
| F10 | Y | N | 60 | 51 | Y | 18 | 22 | 11 | 12 |  |
| G09 | Y | N | 60 | 49 | Y | 12 | 8 | 15 | 5 |  |
| G11 | Y | N | 60 | 60 | Y | 9 | 6 | 8 | 2 |  |
| **Total Inside Perimeter:** | | | | | | 325 | 273 | 172 | 45 |  |
| **Total Outside Perimeter:** | | | | | | 370 | 199 | 130 | 39 |  |

**Supplementary Table 4:** Results of t-tests of movement metrics for female deer outside the burn perimeter before and after the Mendocino Complex Fire.

| ***Movement Metric*** | ***Pre-fire mean ± SD*** | ***Post-fire mean ± SD*** | ***df*** | ***t*** | ***p-value*** |
| --- | --- | --- | --- | --- | --- |
| Home range size (km^2^) | 0.135 ± 0.097 | 0.200 ± 0.140 | 4 | -1.494 | 0.210 |
| Turn angle correlation | 0.508 ± 0.0133 | 0.500 ± 0.0321 | 4 | 0.607 | 0.577 |
| Daily movement distance (km) | 1944 ± 1358 | 1807 ± 358 | 4 | 0.281 | 0.792 |
| Mean residence time (min) | 17.557 ± 9.754 | 13.843 ± 5.892 | 4 | 0.753 | 0.493 |
| Mean time to return (min) | 46.837 ± 12.116 | 48.646 ± 12.840 | 4 | -0.324 | 0.763 |
| Straightness index | 0.012 ± 0.007 | 0.012 ± 0.005 | 4 | 0.192 | 0.857 |

**Supplementary Table 5:** Model selection for RSF models for deer (pre- and post-fire) at the Hopland Research and Extension Center. Table includes all candidate models explored in the backwards stepwise selection process, as well as the null models (intercept and random effect only). The best (full) model is bolded, and models within 2 AIC are italicized. K = number of model parameters. AIC = Akaike Information Criterion. ∆AIC = difference between model AIC and the AIC of the best model.

| ***Pre-Fire Models*** | | |
| --- | --- | --- |
| **Model** | **AIC** | **∆AIC** |
| **Veg Type + Elevation + Ruggedness + Slope + Aspect (Eastness) + Aspect (Northness) + Distance to Streambed + (1\|Animal ID)** | **30106** | **0** |
| *Veg Type + Elevation + Ruggedness + Slope + Aspect (Eastness) + Aspect (Northness) + Distance to Streambed + Sex + (1\|Animal ID)* | *30107* | *1* |
| Veg Type + Elevation + Slope + Aspect (Eastness) + Aspect (Northness) + Distance to Streambed + (1\|Animal ID) | 30109 | 3 |
| Veg Type + Elevation + Slope + Aspect (Eastness) + Aspect (Northness) + Distance to Streambed + Sex + (1\|Animal ID) | 30111 | 5 |
| Veg Type + Elevation + Ruggedness + Slope + Aspect (Eastness) + Aspect (Northness) + (1\|Animal ID) | 30117 | 11 |
| Veg Type + Elevation + Ruggedness + Slope + Aspect (Eastness) + Aspect (Northness) + Sex + (1\|Animal ID) | 30118 | 12 |
| Elevation + Ruggedness + Slope + Aspect (Eastness) + Aspect (Northness) + Distance to Streambed + (1\|Animal ID) | 30122 | 16 |
| Elevation + Ruggedness + Slope + Aspect (Eastness) + Aspect (Northness) + Distance to Streambed + Sex + (1\|Animal ID) | 30123 | 17 |
| Veg Type + Ruggedness + Slope + Aspect (Eastness) + Aspect (Northness) + Distance to Streambed + (1\|Animal ID) | 30139 | 33 |
| Veg Type + Ruggedness + Slope + Aspect (Eastness) + Aspect (Northness) + Distance to Streambed + Sex + (1\|Animal ID) | 30141 | 35 |
| Veg Type + Elevation + Ruggedness + Slope + Distance to Streambed + (1\|Animal ID) | 30162 | 56 |
| Veg Type + Elevation + Ruggedness + Slope + Distance to Streambed + Sex + (1\|Animal ID) | 30164 | 58 |
| Veg Type + Elevation + Ruggedness + Aspect (Eastness) + Aspect (Northness) + Distance to Streambed + (1\|Animal ID) | 30165 | 59 |
| Veg Type + Elevation + Ruggedness + Aspect (Eastness) + Aspect (Northness) + Distance to Streambed + Sex + (1\|Animal ID) | 30167 | 61 |
| Null: (1\|Animal ID) | 30303 | 197 |
| ***Post-Fire Models*** | | |
| **Model** | **AIC** | **∆AIC** |
| **Veg Type + Elevation + Ruggedness + Slope + Aspect (Eastness) + Aspect (Northness) + Distance to Streambed + Surv Veg + (1\|Animal ID)** | **26151** | **0** |
| *Veg Type + Elevation + Ruggedness + Slope + Aspect (Eastness) + Aspect (Northness) + Distance to Streambed + Surv Veg + Sex + (1\|Animal ID)* | *26152* | *1* |
| Veg Type + Elevation + Ruggedness + Aspect (Eastness) + Aspect (Northness) + Distance to Streambed + Surv Veg + (1\|Animal ID) | 26158 | 7 |
| Veg Type + Elevation + Ruggedness + Aspect (Eastness) + Aspect (Northness) + Distance to Streambed + Surv Veg + Sex + (1\|Animal ID) | 26160 | 9 |
| Veg Type + Elevation + Ruggedness + Slope + Aspect (Eastness) + Aspect (Northness) + Surv Veg + (1\|Animal ID) | 26162 | 11 |
| Veg Type + Elevation + Ruggedness + Slope + Aspect (Eastness) + Aspect (Northness) + Surv Veg + Sex + (1\|Animal ID) | 26164 | 13 |
| Veg Type + Ruggedness + Slope + Aspect (Eastness) + Aspect (Northness) + Distance to Streambed + Surv Veg + (1\|Animal ID) | 26190 | 39 |
| Veg Type + Ruggedness + Slope + Aspect (Eastness) + Aspect (Northness) + Distance to Streambed + Surv Veg + Sex + (1\|Animal ID) | 26192 | 41 |
| Veg Type + Elevation + Ruggedness + Slope + Distance to Streambed + Surv Veg + (1\|Animal ID) | 26212 | 61 |
| Veg Type + Elevation + Ruggedness + Slope + Distance to Streambed + Surv Veg + Sex + (1\|Animal ID) | 26214 | 63 |
| Veg Type + Elevation + Slope + Aspect (Eastness) + Aspect (Northness) + Distance to Streambed + Surv Veg + (1\|Animal ID) | 26261 | 110 |
| Veg Type + Elevation + Slope + Aspect (Eastness) + Aspect (Northness) + Distance to Streambed + Surv Veg + Sex + (1\|Animal ID) | 26263 | 112 |
| Veg Type + Elevation + Ruggedness + Slope + Aspect (Eastness) + Aspect (Northness) + Distance to Streambed + (1\|Animal ID) | 26566 | 415 |
| Veg Type + Elevation + Ruggedness + Slope + Aspect (Eastness) + Aspect (Northness) + Distance to Streambed + Sex + (1\|Animal ID) | 26568 | 417 |
| Elevation + Ruggedness + Slope + Aspect (Eastness) + Aspect (Northness) + Distance to Streambed + Surv Veg + (1\|Animal ID) | 26760 | 609 |
| Elevation + Ruggedness + Slope + Aspect (Eastness) + Aspect (Northness) + Distance to Streambed + Surv Veg + Sex + (1\|Animal ID) | 26762 | 611 |
| Null: (1\|Animal ID) | 27763 | 612 |

**Supplementary Table 6:** Model selection for Body Condition Index of deer (female and male) at the Hopland Research and Extension Center. Table includes all candidate models explored in the selection process. The top models are bolded. AIC = Akaike Information Criterion. ∆AIC = difference between model AIC and the AIC of the best model.

| ***Female deer*** | | |
| --- | --- | --- |
| **Model** | **AIC** | **∆AIC** |
| **Burn Area * Time Period + (1\|Camera)** | **2940** | **0** |
| Time Period + (1\|Camera) | 2945 | 5 |
| Days Since Fire + (1\|Camera) | 2972 | 32 |
| Days Since Fire * Burn Area + (1\|Camera) | 2977 | 37 |
| Null: (1\|Camera) | 2996 | 56 |
| Burn Area + (1\|Camera) | 2998 | 58 |
| ***Male deer*** | | |
| **Model** | **AIC** | **∆AIC** |
| **Null: (1\|Camera)** | **951** | **0** |
| Burn Area + (1\|Camera) | 954 | 3 |
| Days Since Fire + (1\|Camera) | 956 | 5 |
| Time Period + (1\|Camera) | 956 | 5 |
| Burn Area * Time Period + (1\|Camera) | 962 | 11 |
| Days Since Fire * Burn Area + (1\|Camera) | 964 | 13 |

**Supplementary Table 7:** Beta-coefficients and standard errors for all coefficients in the top models for male and female deer Body Condition Index at the Hopland Research and Extension Center. The reference level for Burn Area was Outside, and for Time Period was Pre-Fire. All models included camera trap as a random effect.

| ***Female deer*** | | |
| --- | --- | --- |
| **Variable** | *Beta Coefficient* | *Standard Error* |
| Intercepts | 3.039 | 0.139 |
| Burn Area = Inside | -0.145 | 0.079 |
| Time Period = Post-Fire | -0.237 | 0.165 |
| (Burn Area = Inside) * (Time Period = Post-Fire) | -0.340 | 0.108 |
| ***Male Deer*** |  |  |
| Intercept | 3.634 | 0.115 |

**Supplementary Figure 1**. Locations of camera traps in the Hopland and Research Extension Center. Only cameras that were included in the deer Body Condition Index analysis are shown on the map (see Supplementary Table 3).
